# Dissociable use-dependent processes for volitional goal-directed reaching

**DOI:** 10.1098/rspb.2022.0415

**Published:** 2022-04-27

**Authors:** Jonathan S. Tsay, Hyosub E. Kim, Arohi Saxena, Darius E. Parvin, Timothy Verstynen, Richard B. Ivry

**Affiliations:** ^1^ Department of Psychology, University of California, Berkeley, USA; ^2^ Helen Wills Neuroscience Institute, University of California, Berkeley, USA; ^3^ Department of Physical Therapy, University of Delaware, Newark, DE, USA; ^4^ Department of Psychological and Brain Sciences, University of Delaware, Newark, DE, USA; ^5^ Department of Psychology, Carnegie Mellon University, Pittsburgh, PA, USA

**Keywords:** use-dependent learning, action selection, action execution, reinforcement learning, reward

## Abstract

Repetition of specific movement biases subsequent actions towards the practiced movement, a phenomenon known as use-dependent learning (UDL). Recent experiments that impose strict constraints on planning time have revealed two sources of use-dependent biases, one arising from dynamic changes occurring during motor planning and another reflecting a stable shift in motor execution. Here, we used a distributional analysis to examine the contribution of these biases in reaching. To create the conditions for UDL, the target appeared at a designated ‘frequent’ location on most trials, and at one of six ‘rare’ locations on other trials. Strikingly, the heading angles were bimodally distributed, with peaks at both frequent and rare target locations. Despite having no constraints on planning time, participants exhibited a robust bias towards the frequent target when movements were self-initiated quickly, the signature of a planning bias; notably, the peak near the rare target was shifted in the frequently practiced direction, the signature of an execution bias. Furthermore, these execution biases were not only replicated in a delayed-response task but were also insensitive to reward. Taken together, these results extend our understanding of how volitional movements are influenced by recent experience.

## 1. Introduction

Repetition can bias future movements to resemble recently repeated ones [1], a phenomenon referred to as use-dependent learning (UDL). The effects of UDL can be seen in features such as the direction and speed of the current movement, and has been observed in movements ranging from single-joint actions to whole-body locomotion [2–10]. Theoretically, these movement biases have been attributed to shifts in the tuning of neurons towards the direction of a frequently practiced movement, a form of plasticity that alters the sensorimotor map underlying movement execution [11–15].

Recent work has challenged this perspective, suggesting that movement biases also stem from limitations associated with motor planning [16]. That is, when preparing to act, participants may generate (or maintain) a default plan associated with the practiced movement and have to override this plan when the context requires a different action. This hypothesis predicts that planning biases should be most pronounced when preparation time is limited and become markedly reduced when preparation time is long. Indeed, residual biases observed when planning time is long can provide an estimate of use-dependent effects on action execution. This hypothesis has received support from experiments that impose tight constraints on planning time: Marinovic *et al.* [17] asked participants to make isometric forearm contractions to move a cursor to a visual target that appeared at either a frequent location or one of several rare locations. Using a timed response task [18], participants were provided with either a short (150 ms) or long (500 ms) planning period. Movement biases were large when preparation time was short, and small when preparation time was long, dynamics which suggest the influence of use-dependent biases on motor planning. However, a small bias persisted even in the long preparation condition, an effect that can be viewed as the signature of an execution bias.

In the present study, we set out to further examine the sources of UDL, asking if these dissociable forms of bias would be manifest when action planning and execution occur as the movements are produced without any overt temporal constraints on movement initiation. Under such conditions, volitional movement may be initiated only after the competition between different motor plans is resolved [19–22]. By this view, only execution-based biases should be observed in an unpressured environment. Alternatively, recent work suggests that movement initiation and preparation may function as two independent processes [23]. By this view, volitional movements could be made prior to the system fully resolving competing motor plans, resulting in both planning and execution biases even in a temporally unpressured context.

To evaluate these hypotheses, participants were instructed to make volitional, self-paced reaching movements to one of seven target locations, without explicit constraints on movement initiation time. To create conditions for UDL, one target appeared on 86.8% of the trials and the other locations on 2.2% of the trials each, a design similar to that used in prior studies of UDL [24,25]. We focused on a distributional analysis of the movement heading angles. Specifically, if both forms of bias are operative, the distributions would be expected to be bimodal: biases associated with motor planning should manifest as a broad distribution of heading angles between the frequent and rare target locations, with larger biases towards the frequent target associated with movements initiated quickly. By contrast, execution biases should manifest as a narrow distribution of heading angles near the rare target location, with the degree of bias insensitive to planning time but instead arising from stable, use-dependent plasticity in the tuning of the sensorimotor map.

While the term ‘use-dependent’ might suggest a form of Hebbian plasticity where the strength of learning is a function of repetition, evidence from both rodent [25,26] and human work indicates that reward can enhance UDL [27,28]. Previous UDL studies in humans, however, have used tasks in which reinforcement was always provided, either in the form of motivational verbal cues [1,17] or points to encourage faster movement initiation and/or movement accuracy [25,27,28]. These reinforcers, intended to enhance participants' motivation, may have biased participants to adopt a default motor plan to maximize reward. We eliminated all explicit forms of reinforcement in Experiment 1 and directly test the impact of reward on UDL in Experiment 2.

## 2. Results

### (a) Experiment 1

Participants performed centre-out reaches, moving to a visual target that appeared at either a frequent location or rarely presented locations. We did not impose any constraint on reaction time (RT), allowing the participants to initiate movements at their own pace. Since individual reaches were composed of straight and curved reaches (electronic supplementary material, figure S4), we extracted the heading angle approximately 40 ms after movement initiation, a time point that should index the participant's initial movement plan prior to any online corrections.

**Figure 1.**
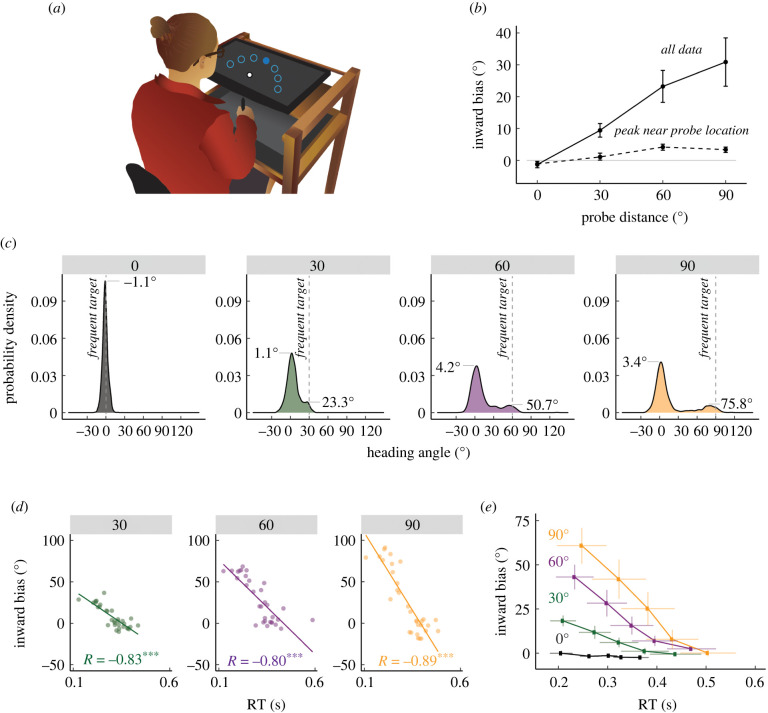
Hasty reaches elicited greater movement biases in Experiment 1. (*a*) Reaching set-up showing locations of frequent and rare probe targets. Only one of seven targets (filled blue circle) was visible on each trial. (*b*) Average inward biases increased as a function of probe distance (solid line). By contrast, the peak of the Gaussian estimated from the distribution near the probe location saturated for larger probe distances (dashed line). (*c*) Distribution of heading angles for each of the probe distances. Dashed line denotes the location of the frequently presented context target, and 0 on the *x*-axis denotes the location of the probe target. The means obtained from the mixture of Gaussians model are provided. (*d*) Bias as a function of a RT for a representative participant. Dots indicate individual reaches with the thin line showing the best-fitting regression line. *R* denotes Pearson correlation; *** = *p* < 0.001. (*e*) Group-level analysis of bias as a function of RT. For each individual, RTs were binned into quintiles and mean bias was calculated for each quintile. These data were then averaged across the group. Error bars denote SEM. (Online version in colour.)

Movement biases towards the repeated location were evident in the reaches made to the probe locations, with the mean heading angle shifted towards the location of the frequent target (figure 1*b*). Averaging across participants and target direction (relative to the frequently repeated location), the size of the bias ranged from an average of 7.6° for the probe closest to the frequent target to 28.2° for the most distant probe location (main effect of probe distance: $\chi_{\left(1\right)}=7909.3,$ $\chi_{\left(1\right)}=7909.3,p<0.001,\eta^{2}=0.5,\chi_{\left(1\right)}=7909.3,$ $\;p<0.001,\eta^{2}=0.5$). Biases were negligible at the frequent target location (1.5±0.7). When tested against the null, no-bias hypothesis, the biases for all of the probe locations were significant (30°: $7.6\;\pm 2.1;\;t_{9}=\;3.7,$ p=0.005,d=1.2; 60°: $19.9\;\pm 4.2;\;t_{9}=\;4.8,p<0.001,$ d=1.5; 90°: 2$8.2\;\pm 6.7;\;t_{9}=\;4.2,p=0.002,\;d=1.3$). Moreover, the magnitude of the bias increased with probe distance (Bonferroni corrected for three comparisons: 30° versus 60°: $12.3\pm 2.7;t_{9}=\;5.2,p=0.002,d=1.6$; 60° versus 90°: $8.2\;\pm 4.3;\;t_{9}=\;2.2,p=0.15,d=0.7$; 30° versus 90°: $20.5\;\pm 5.7;\;t_{9}=\;4.2,p=0.006,d=1.3$).

We evaluated whether these biases reflected an overall shift in the heading angle distribution, a mixture of reaches to the frequent and rare target locations or a combination of these factors. We reasoned that use-dependent changes in tuning profiles should produce an overall shift in the distribution, whereas a mixture of two types of movements would manifest as a bimodal distribution of heading angles. As seen in both the group (figure 1*c*) and individual data (electronic supplementary material, figure S5, top), the data for all probe distances were bimodal (likelihood ratio test of bimodal versus unimodal, probe distance 30°, 60°, 90°: all p<0.001; probe distance 0° is unimodal: p=0.08). There was one peak at the probe location and a second peak near the frequent target location, similar to the results reported in a UDL study involving eye movements [24]. In addition, the distributions included a wide range of intermediate heading angles. As such, the distribution of heading angles exhibited signatures of a dynamic competition between preparatory processes required for reaching the target location yet are biased by the expectation that the target will appear at the frequent location. The intermediate reaches could reflect a plan to reach close enough so that a curved reach path due to a rapid online correction could hit the target [29]. The intermediate angles could also arise from an integration of two distinct plans, or alternatively, an initial default plan to the frequent location that was redirected towards the actual target through an internal transformation, such as mental rotation [30,31]. Regardless of the exact mechanism, these data are consistent with the view that action preparation and initiation operate with some degree of independence [23], with participants sometimes initiating movement prior to the full completion of planning (see electronic supplementary material, Discussion: *Mechanistic accounts of motor planning biases*).

We next examined the relationship between heading angle and RT. Given the bimodal nature of the heading distributions, we reasoned that faster RTs would be associated with (incorrect) movements towards the frequent target location, whereas slower RTs would be associated with (correct) movements towards the rare target. Consistent with this planning time hypothesis, we observed a strong negative relationship between heading angle (bias) and RT. As shown in the data from one representative participant (figure 1*d*), inward biases towards the frequent target were much larger when RT was fast compared to when RT was slow; indeed, there was a cluster of reaches to the frequent target location for the fastest RTs and a cluster of reaches to the rare probe target location for the slowest RTs.

This negative correlation was observed in the individual data for nine of the 10 participants (electronic supplementary material, figure S6) and at the group level for all of the probe distances (bias versus RT slope, 30°: −55.3±3.1; $t_{7191}=-17.8,p<0.001;$ 60°: $-99.1\;\pm 2.9;\;t_{7191}=-34.1,$ p<0.001; 90°: $-136.7\;\pm 2.5;\;t_{7191}=-5.7,p<0.001$). By contrast, this correlation was not observed for probe trials in which the target appeared at the frequent target location (0°: $1.2\;\pm 1.3;\;t_{6965}=0.9,p=0.35$). To visualize this effect, the RT data were segmented into five evenly sized bins (quintiles), with the bias data for each quintile averaged across participants (figure 1*e*). To examine how probe distance impacts the bias–RT relationship, we opted to normalized the bias data within each probe distance to mitigate any spurious differences across these conditions (i.e. the range of inward biases inherently differ with probe distance): we did not find a significant difference in the rates at which biases were attenuated across different probe distances (electronic supplementary material, figure S7, bias × RT interaction: $\chi_{\left(1\right)}=\;3.1,p=0.21,\eta^{2}=0.0$).

These findings motivated us to re-examine the data from the Verstynen & Sabes [25] study given that this paper is frequently cited as an example of use-dependent effects on movement execution (despite the authors themselves being agnostic about whether biases arise from motor execution or planning). Consistent with the results of the current experiment, the heading angles in their data were broadly distributed and showed two peaks, one near the rare target location and the other near the frequent target locations (see electronic supplementary material, result I; figures S1 and S2). Moreover, their data also showed a strong association between the bias and RT, with faster RTs associated with greater biases and slower RTs associated with smaller biases. These three signatures—bimodality, broad range of heading angles and the negative correlation between RT and bias—reveal a strong contribution of planning-based UDL in the Verstynen & Sabes data.

By employing a single repeated target location and relatively rare probe targets in Experiment 1, we may have artificially encouraged a planning-based bias. It would be reasonable for participants under such conditions to prepare (or maintain across trials) a default movement plan for a reach to the frequent target location rather than re-establish a new movement plan on every trial. Moreover, although we did not provide an extrinsic reward (e.g. points or a positive tone) to reinforce movements to the frequent target location, the visual feedback may have elicited intrinsic reinforcement based on whether or not the cursor intersected the target [32–34]. This feedback might have disproportionately reinforced the plan associated with the frequent target. These concerns led us to conduct a supplemental experiment in which participants were assigned to one of three groups that differed in terms of the distribution of the frequent target location: fixed frequent target location (as in Experiment 1, s.d. = 0°), or from a normal distribution with a standard deviation of either 7.5° or 15°. We also removed all visual feedback to minimize any effect of intrinsic reward. We again observed bimodality in the heading angle data, especially for the larger probe distances, and a strong modulatory effect of RT on bias (electronic supplementary material, result II; figure S3).

Overall, the results of Experiment 1, our re-analysis of Verstynen & Sabes, and the supplemental experiment indicate that a large component of use-dependent biases originate from limitations in action planning. Nonetheless, there may also be a bias component associated with subtle changes in motor execution. To examine this hypothesis, we reasoned that the peak of the Gaussian centred around the rare locations could provide a rough estimate of an execution bias, under the assumption that this inferred distribution is composed of reaches directed (planned) towards the actual target. Indeed, these peaks were shifted in the direction of the frequent target location for each of the three probe distances in Experiment 1 (95% bootstrapped confidence interval from 10 000 samples is greater than 0°, the rare target location: 30°: [0.1°, 2.3°], 60°: [3.2°, 5.1°], 90°: [2.5°, 4.2°]). A similar pattern was found in our supplemental experiment and our re-analysis of Verstynen & Sabes. Together, these results are consistent with the hypothesis that repeated movements result in a small execution bias. However, this bias could also reflect residual planning-related activity; that is, the peak near the probe location may be contaminated by the inclusion of some reaches that involved the blending of movement plans or transformation of a default plan, one that is just approaching the actual target location. This ambiguity motivated us to modify the experimental task in Experiment 2 to provide a more direct assessment of execution-based UDL.

### (b) Experiment 2

The results of Experiment 1, our re-analysis of Verstynen & Sabes [25], and the supplemental experiment all point to the presence of two sources of bias, one associated with motor planning and a second associated with motor execution (see [17]). The distribution of heading angles observed in these reaching tasks is similar to that observed in prior studies involving either isometric forearm movements [17] or eye movements [24]. Interestingly, this similarity holds even though planning time was experimentally manipulated in the forearm and eye movement studies, whereas in our study, there were no explicit constraints imposed on RT. However, our participants may operate under a self-imposed urgency signal [35,36], one primed by the experimental instructions to ‘reach as quickly and accurately as possible’ (i.e. constraints on movement time) or a desire to complete the experiment as fast as possible.

Our analysis of the execution component was indirect, inferred by the peak of the Gaussian around the probe location in the mixture of Gaussians model. As a more direct test, we used a delayed-response task in Experiment 2, imposing a 500 ms delay between the presentation of the target location and imperative signal [17,24]. We reasoned that the additional preparation time would allow participants to complete planning processes in advance of the imperative and thus allow us to estimate use-dependent biases in movement execution with minimal contamination from use-dependent biases in motor planning.

We also used Experiment 2 to examine the influence of reward on execution-based UDL, comparing the performance of a group who received binary, rewarding feedback following accurate movements to the frequent target (Reward group) to a group who received no feedback (No Reward group). Importantly, unlike most studies that include some form of extrinsic reward to incentivize task success [17,24,25], the No Reward group represents a ‘pure’ use-dependent condition as they never received any type of reinforcement (similar to: [24]), including any potential intrinsic reward from seeing a cursor hit the target [24,32,37].

**Figure 2.**
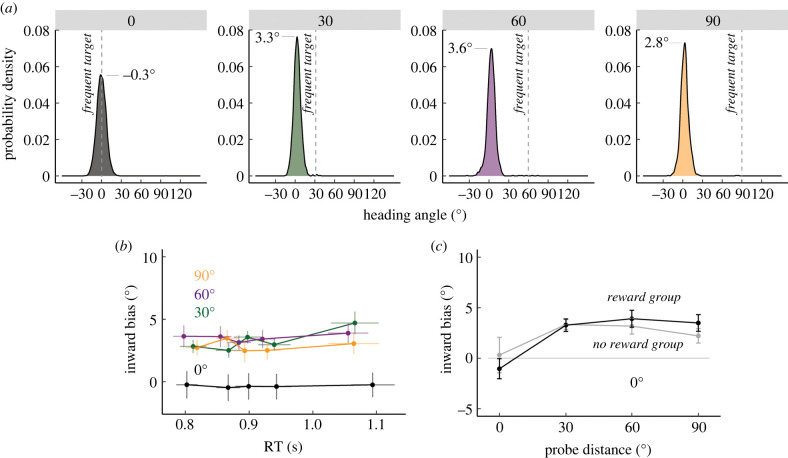
Use-dependent motor execution biases were small and not modulated by reward in Experiment 2. (*a*) Distribution of heading angles for each of the probe distances. Dashed line denotes the location of the frequently presented context target, and 0 on the *x*-axis denotes the location of the probe target. The means obtained from the mixture of Gaussian model are provided. (*b*) Group-level quintile analysis of bias versus RT. Reward and No Reward groups were combined in this panel since neither RTs nor movement biases varied with reward feedback. Error bars denote SEM. (*c*) Average inward biases were modest for both Reward and No Reward groups. (Online version in colour.)

We first asked whether biases persist when planning time is extended. The distributional analysis showed a marked difference from that observed in Experiment 1. The distribution of heading angles for all probe distances (in both groups) was tightly clustered around the rare target location, with minimal heading angles in the direction of the frequent target location (figure 2*a*). Indeed, the distributions were all best described as unimodal (likelihood ratio test of bimodal versus unimodal, all probe distances: p=1), with a single peak near the location at which the probe target appeared. Although the delay yielded unimodal distributions, the peaks of the distributions were not centred at the probe locations but instead consistently shifted in the direction of the frequent target location (95% bootstrapped confidence interval from 10 000 samples: 30°: [3.0°, 3.7°], 60°: [3.3°, 3.9°], 90°: [2.5°, 3.2°]).

This persistent, albeit small, bias towards the frequent target for all probe distances was corroborated by a series of post hoc *t*-tests (30°: $3.3\;\pm 0.4;\;t_{31}=\;8.6,p<0.001,$ d=1.5; 60°: $3.5\;\pm 0.6;\;t_{31}=\;6.2,p=0.001,d=1.1$; 90°: 2.8$\;\pm \;0.6;t_{31}=\;5.2,p<0.001,d=0.9$). The bias was not present for reaches to the frequent target location (0°: $-0.4\;\pm 0.6;t_{31}=\;-0.4,p=0.72,d=0.1$). Moreover, the magnitude of the bias did not scale with probe distance (Bonferroni corrected for three comparisons: 30° versus 60°: $0.2\;\pm 0.5;t_{31}=0.5,p=1,d=0.1$; 60° versus 90°: $0.7\;\pm 0.4;t_{31}=1.6,p=0.33,d=0.3$; 30° versus 90°: $0.5\;\pm 0.5;\;t_{31}=\;0.9,p=1,d=0.2$) and was not (negatively) correlated with RT for any of the probe distances (figure 2*b*) (RT versus bias slope, 30°: 4.2 ±1.1; $t_{24726}=3.7,p<0.001;$ 60°: $1.6\;\pm 1.3,t_{24723}=1.2,p=0.24$; 90°: −1.6±0.9, $t_{24723}=$ −1.7,p=0.09). Curiously, there was a small positive correlation for probe distance 30°, with the bias slightly larger on trials with longer RTs (figure 2*b*). While the cause of the positive slope is unclear, the effect was modest (4.2°/s) in comparison to values observed in Experiment 1 and Verstynen & Sabes data (range between −55.3°/s and −244.5°/s).

We next examined the influence of reward on the small biases observed across probe targets. To evaluate if the reward manipulation was effective, we compared movement accuracy between the two groups for reaches to the frequent target (the only location eligible for reward in the Reward group). As shown in the cumulative distribution functions (electronic supplementary material, figure S9), participants in the Reward group were more accurate than those in the No Reward group ($t_{30}=\;3.2,p=0.003,d=1.1$), confirming that our reward manipulation had an appreciable effect on behaviour. Importantly, the reward manipulation had no effect on execution biases (figure 2*c*) (absent main effect of reward: $0.1\pm 0.9;\;\chi_{\left(1\right)}=\;0,p=0.92,\eta^{2}=0.02$; absent reward × probe distance interaction: $\chi_{\left(1\right)}=\;4.3,p=0.12,\eta^{2}=0$).

Taken together, the results of Experiment 2 highlight three points: first, the large biases observed in Experiment 1 were minimized by the introduction of a short delay period between the presentation of the target and the imperative signal, similar to what has been observed in experiments using a timed RT task in which preparation time is extended [17,24]. This attenuation reinforces the idea that the main source of bias in Experiment 1 arose from limitations in movement planning. Second, the small bias observed in Experiment 2 has the expected signatures of a use-dependent change in the sensorimotor mapping underlying movement execution, namely that the effect is temporally stable and invariant with respect to RT. Third, this stable bias was not modulated by the presence of reward. One interpretation of this null result is that execution-based UDL operates independently of processes involved in reinforcement learning. Alternatively, the null result might reflect a ceiling effect: the magnitude of the bias did not scale with probe distance, suggesting that there may be an upper bound on the degree of short-term plasticity in this form of UDL, one that is not elevated by reward.

## 3. Discussion

UDL refers to the phenomenon in which movements are biased to resemble recently repeated movements. This term was initially introduced to capture an implicit form of plasticity in which repetition shifts the tuning of the sensorimotor map in the direction of a frequently practiced movement [1,3]. However, subsequent work has led to a broadening of the term to include biases from processes associated with motor planning [17,24]. These planning-related biases are especially pronounced under conditions in which planning time is limited [29].

In the present study, we further explored the contribution of planning—and execution-based UDL, employing a simple reaching task in which participants were free to initiate each movement of their own volition. We observed distinct signatures of planning and execution biases. In Experiment 1, planning-related bias was inferred from the distribution of heading angles and the relationship between heading angle and RT. When cued to reach to a rare probe target, the distributions were frequently bimodal, with a large peak near the actual target location and a smaller peak near the frequent target location. Thus, the mean bias scaled with the distance between the probe location and the frequent target location. Moreover, in all conditions, the distributions were broad, encompassing initial heading angles that spanned the range between the frequent target location and the actual target location. Strikingly the magnitude of the bias was strongly correlated with RT, with the largest bias observed for the shortest RTs (see electronic supplementary material, Discussion: Mechanistic accounts of motor planning biases).

By contrast, execution-based bias was inferred from the peak of the heading angle distributions for reaches to the probe locations. This peak was shifted slightly in the direction of the frequent target location and did not scale with probe distance. Importantly, our estimate of the magnitude of this bias was comparable in a delayed-response task (Experiment 2), a manipulation introduced to minimize the contribution of planning-related bias. Unlike planning-related bias, execution-based UDL did not vary with RT in Experiment 2.

This two-process account of UDL is consistent with previous work, including studies using isometric forearm movements [17] and saccadic eye movements [24]. In previous work, the planning-based component manifested in conditions in which the experimental instructions were designed to limit planning time [17,24] or reward was based in part on fast RTs [25]. As shown in the present work, these experimental manipulations are not required. However, we also recognize that the participants in Experiment 1 (and the electronic supplementary experiment) may have operated under a self-imposed deadline (e.g. to complete the experiment quickly) or have been biased to initiate each movement quickly given the instructions that emphasize fast movement speed. Intermediate reaches could reflect either an ‘optimal’ plan to reach close enough so that a curved reach path due to a rapid online correction could hit the target [29], an integration of two distinct plans (i.e. response substitution) [38], or alternatively, an initial default plan to the frequent location that was redirected towards the actual target through an internal transformation, such as mental rotation [30,31].

Execution-based UDL has been attributed to subtle changes in the tuning of the sensorimotor map as a function of repetition. While we cannot definitively rule out whether our estimate of this bias includes some residual attraction in planning space, the absence of any dependency on RT suggests that this form of bias reflects a stable form of plasticity. Interestingly, our estimate of the magnitude of this bias based on the distributional analyses presented in the current paper is similar to those observed in previous studies (electronic supplementary material, figure S10), despite the variety of effectors and tasks employed. The fact that this form of learning does not scale with probe distance (at least within the range of probe distances tested here) is puzzling. It may be that this form of UDL is based on a Hebbian-like mechanism [25] involving the potentiation of locally tuned units with broad basis functions [39], one that rapidly saturates, and only decreases when absolute probe distances go beyond 90° [17,24,39]. We note that saturation is also observed in implicit, error-based learning (i.e. sensorimotor adaptation) [40–47], although the magnitude in retuning based on error is considerably greater than that obtained from repetition.

The absence of an effect of reward on execution-based UDL is surprising given that signals related to reward enhance plasticity effects in the motor cortex [48–50]. For example, the change in bias of a movement elicited by suprathreshold TMS toward the direction of a recently practiced movement is enhanced by a dose of levodopa [51]. Given that there is no planning of these TMS-elicited movements, the bias presumably comes about from transient changes in neural tuning and these changes are boosted by the levodopa. This raises the question of why TMS-induced biases may be boosted by reward whereas biases from the active movement in Experiment 2 are not. As corticospinal neurons that are sensitive to dopamine have been shown to have a higher resting potential [52,53], we speculate that the widespread and highly synchronized discharge of neurons induced by the TMS pulse may disproportionately activate these reward-sensitive neurons. By contrast, active movements likely entail the recruitment of a broader range of neurons relevant for the planned action, diluting the putative effect of reward [54].

While our results suggest that reward does not modulate residual UDL biases in action execution, it is well established that reward modulates biases associated with action planning [55–57]. Mechanistically, reinforcement is thought to enhance movement preparation and vigour towards a reinforced location (i.e. the frequent target). That is, the weight given to the movement plan towards a reinforced target increases with the amount of reward and thus more time is required to overcome this bias when the target appears at a rare location. This last point is relevant when considering the contribution of UDL to skill acquisition. While the benefits of extended practice have been attributed to long-term re-organization in primary motor cortex [58], practice-related changes surely impact upstream premotor and sensory areas. Similar to the results of the current study, the benefits of long-term practice may reflect flexible processes involved in motor planning as well as small use-dependent changes associated with motor execution [59,60].

## 4. Methods

### (a) Participants

A total of 42 participants (mean age = 20 ± 2.2 years) were recruited for two experiments. The sample sizes were based on similar reaching studies assessing UDL (Marinovic *et al*. [17]; Verstynen & Sabes [25]). All participants were right-handed as verified with the Edinburgh Handedness Inventory [61] and received course credit or financial compensation for their participation. The experimental protocol was approved by the Institutional Review Board at the University of California, Berkeley.

### (b) Reaching task

The participant was seated at a custom-made table that housed a horizontally mounted LCD screen (53.2 cm by 30 cm, ASUS), positioned 27 cm above a digitizing tablet (49.3 cm by 32.7 cm, Intuos 4XL; Wacom, Vancouver, WA) (figure 1*a*). Stimuli were projected onto the LCD screen. The experimental software was custom written in Matlab using the Psychtoolbox extensions [62].

Participants made centre-out reaching movements, sliding a modified air hockey paddle containing an embedded digitizing stylus across the tablet. The tablet recorded the position of the stylus at 200 Hz. Vision of the hand was blocked by the monitor, and the lights in the room were turned off to block peripheral vision of the arm.

At the beginning of each trial, participants moved their right hand to position the digitizing pen within a ‘start’ circle (0.6 cm diameter open white circle in the centre of the LCD screen). To assist the participant in finding this starting position, a white feedback cursor (0.5 cm diameter) appeared when the hand was within 2 cm of the start circle. The position of the cursor was aligned with the digitizing pen. Once the pen remained within the start circle for 500 ms, the target appeared (blue circle, 1 cm diameter). The radial position of the target was always 10 cm from the start circle. In terms of angular position, the target could appear at one of seven locations, 0°, 30°, 60°, 90°, 120°, 150° and 330° (figure 1*a*).

Participants were instructed to reach towards the target, making the movement in a smooth, rapid manner. To discourage online corrections, participants were told to slice through the target rather than attempt to stop at the target. A trial ended when the reach amplitude exceeded 10 cm or when the movement time exceeded 400 ms. Cursor feedback was presented throughout the first 10 cm of the movement trajectory and then remained visible at the target radius for 50 ms before turning off.

### (c) Experiment 1

Ten participants were tested in Experiment 1. To create the conditions for UDL, the target appeared at one location with a much higher probability than at the other six locations (86.8% versus 2.2% for each of the other six locations). For half of the participants, the frequent target location was 60°; for the other half of the participants, the frequent target location was at 150°.

Each participant completed two baseline blocks and eight test blocks. In the first baseline block, the target appeared 10 times at each of the seven locations. Cursor feedback was presented during the reach. The second baseline block consisted of another 10 reaches to the seven target locations, but no feedback was presented during the reach (or at the endpoint), allowing an estimate of each participant's idiosyncratic biases.

The main experiment consisted of eight test blocks of 90 trials each. Following the design of Verstynen & Sabes [25], each block started with 10 reaches to a target appearing at the high probability location to clearly establish this as the frequent target. This was followed by 80 more trials. Of these, the target appeared at the frequent location on 66 trials and feedback was provided during the reach. For the other 14 trials, the target appeared at one of the seven locations (including the frequent location) and the reaches were made without feedback. We did not provide feedback on these 14 probe trials to ensure that the participant was unaware of his or her bias. The order of the last 80 trials in each block was pseudorandomized, such that there was one probe trial every seven reaches.

The instructions emphasized that the reaches should be made quickly and in one smooth motion, attempting to intersect the target. To discourage movement speeds that might allow for online corrections, the message ‘too slow’ was played over the computer speaker when movement time was greater than 400 ms. The error message, ‘too fast’ was played over the computer speaker if the participant initiated the within 70 ms of target onset, a criterion set to eliminate anticipatory movements. Unlike previous studies of UDL, we did not place any restrictions on RT [17,23,24] or incentivize fast RTs with extrinsic reward (i.e. more points for faster RTs) [25]; the participants initiated the movements at their own pace.

### (d) Experiment 2

The general procedure was the same as in Experiment 1, with three exceptions. First, we modified the session structure in Experiment 2. The experiment began with the same two baseline blocks, one with online cursor feedback (70 trials) and one with no feedback (70 trials). This was followed by six test blocks of 134 trials. Within each test block, there were 113 reaches to the frequent target with potential reward feedback (see below) provided and 21 reaches to all seven locations with no feedback provided (three trials/target, probe trials).

Second, we imposed a 500 ms delay between target appearance and an imperative cue, a tone played over the computer loudspeaker. By providing a 500 ms interval to prepare the movement, we sought to reduce or eliminate temporal constraint (either experimenter- or participant-imposed) on action planning.

Third, we eliminated the online cursor feedback (other than during the first baseline block) so that we could examine the impact of reward on movement biases. Participants were randomly assigned to one of the two groups (*n* = 16/group), a Reward group and a No Reward group. For the Reward group, reaches to the frequent target were rewarded when the hand angle at the target amplitude was within ± 5.7° of the target. On these trials, the target turned green, doubled in size (to 2 cm in diameter), and a pleasant ‘ding’ was played. If the hand angle was greater than ± 5.7° of the frequent target, no visual or auditory feedback was provided. For the No Reward group, no feedback was provided on any of the reaches.

### (e) Data analysis

Hand angle was defined as the angle between a line from the start position to the target and a line from the start position to the hand position, measured at 40 ms after movement onset (corresponding to a movement distance of 2.4 ± 0.1 cm; roughly 25% to the target distance). By taking the hand angle at 40 ms, we should eliminate any contamination from online corrections.

We considered two types of bias. First there is the bias to reach to a given target independent of the effects of the experimental manipulation. For each target location, we determined the participant's baseline bias as the mean angular deviation during the baseline no-feedback block. This value should reflect any bias associated with regression to the mean (i.e. reaches to the centre of the workspace) rather than biases due to repeated reaches to a frequently presented target. This value was thus subtracted from each reach to the corresponding target (both frequent and rare targets) in the training block. Second, to calculate use-dependent bias, the frequent target (60° or 150°) was reset to 0° and the other six targets were defined with respect to the frequent target (±30°, 60° or 90°). The sign of the biases was flipped, such that positive values corresponded to biases towards the frequent target (i.e. inward bias) and negative values corresponded to biases away from the frequent target.

These biases were evaluated using a linear mixed effect model (R: lmer function) with RT and probe target distance as fixed (interacting) factors and participant as a random factor [63]. Satterthwaite's degrees of freedom was also provided [64]. Post hoc *t*-tests on the betas from the linear mixed effect model (i.e. main effect of RT, probe distance and interaction of RT × probe distance) were evaluated using emmeans and ANOVA functions in R. The distribution of these biases (heading angles) was also modelled using a mixture of either one or two Gaussians (R: Mclust). A likelihood ratio test was used to discern which of these two distributions provided a better fit to the distribution (R: mclustBootstrapLRT).

RT was defined as the position of the hand when hand velocity exceeded 3 cm s^−1^. We binned each participant's RTs into quintiles, with the first quintile composed of the fastest 20% of reaches and the fifth quintile composed of the slowest 20% of reaches. For each quintile, we calculated the mean bias. The mean RT and bias data for each quintile were then averaged across participants.

All post hoc *t*-tests were two-tailed and Bonferroni corrected for multiple comparisons. Standard effect sizes are reported ($\eta^{2}$ for fixed factors; Cohen's *d*_z_ for within-subjects *t*-tests, Cohen's *d* for between-subjects *t*-tests, Pearson correlation R for linear regression) [65].

## Data Availability

Data and code are available from the Dryad Digital Repository: https://doi.org/10.6078/D1MX4P [66].
